# Presenting features of newly diagnosed rheumatic heart disease patients in Mulago Hospital: a pilot study

**DOI:** 10.5830/CVJA-2012-076

**Published:** 2013-03

**Authors:** Charles Mondo, Charles Musoke, James Kayima, Jurgen Freers, Wanzhu Zhang, Emmy Okello, Barbara Kakande, Wilson Nyakoojo

**Affiliations:** Department of Medicine, Division of Cardiology, College of Health Sciences, Makerere University, Kampala, Uganda; Department of Medicine, Division of Cardiology, College of Health Sciences, Makerere University, Kampala, Uganda; Department of Medicine, Division of Cardiology, College of Health Sciences, Makerere University, Kampala, Uganda; Department of Medicine, Division of Cardiology, College of Health Sciences, Makerere University, Kampala, Uganda; Uganda Heart Institute and Department of Medicine, College of Health Sciences, Makerere University, Kampala, Uganda; Uganda Heart Institute and Department of Medicine, College of Health Sciences, Makerere University, Kampala, Uganda; Uganda Heart Institute and Department of Medicine, College of Health Sciences, Makerere University, Kampala, Uganda; Uganda Heart Institute, Mulago Hospital, Kampala, Uganda

**Keywords:** rheumatic heart disease, clinical presentation, newly diagnosed

## Abstract

**Introduction:**

Rheumatic heart disease (RHD) continues to cause gross distortions of the heart and the associated complications of heart failure and thromboembolic phenomena in this age of numerous high-efficacy drugs and therapeutic interventions. Due to the lack of contemporary local data, there is no national strategy for the control and eradication of the disease in Uganda. This study aimed to describe the presenting clinical features of newly diagnosed patients with RHD, with particular reference to the frequency of serious complications (atrial fibrillation, systemic embolism, heart failure and pulmonary hypertension) in the study group.

**Methods:**

One hundred and thirty consecutive patients who satisfied the inclusion criteria were recruited over a period of eight months from June 2011 to January 2012 at the Mulago Hospital, Uganda. Data on demographic characteristics, disease severity and presence of complications were collected by means of a standardised questionnaire.

**Results:**

Seventy-one per cent of the patients were female with a median age of 33 years. The peak age of the study group was 20 to 39 years, with the commonest presenting symptoms being palpitations, fatigue, chest pain and dyspnoea. The majority of the patients presented with moderate-to-severe valvular disease. Pure mitral regurgitation was the commonest valvular disease (40.2%), followed by mitral regurgitation plus aortic regurgitation (29%). Mitral regurgitation plus aortic regurgitation plus mitral stenosis was found in 11% of patients. There was only one case involving the tricuspid valve. The pulmonary valves were not affected in all patients; 45.9% of patients presented in severe heart failure in NYHA class III/IV, 53.3% had pulmonary hypertension, 13.9% had atrial fibrillation and 8.2% had infective endocarditis. All patients presented with dilated atria (> 49 mm).

**Conclusion:**

A significant proportion of RHD patients present to hospital with severe disease associated with severe complications of advanced heart failure, pulmonary hypertension, infective endocarditis and atrial fibrillation. There is a need to improve awareness of the disease among the population, and clinical suspicion in primary health workers, so that early referral to specialist management can be done before severe damage to the heart ensues.

## Abstract

Rheumatic heart disease (RHD) is the most common acquired cardiovascular disease in children and young adults and remains a major public health problem in developing countries.^1^-^3^ Africa has the largest number of children with the disease; in sub-Saharan Africa, over a million children are estimated to suffer from this debilitating and often fatal condition.^4^

Rheumatic heart disease is the result of damage to the heart valves, which occurs after repeated episodes of acute rheumatic fever (ARF). The valves become stretched and scarred and do not move normally, resulting in regurgitation and/or stenosis. If RHD is not diagnosed and managed early, it may result in heart failure and premature death.^5^

Several factors determine the type of lesion and severity of the disease among affected individuals. Genetic susceptibility and environmental factors (low socio-economic status) are the key determinants of disease pathogenesis.^1^-^4^ The extent to which environmental factors impact on the pathogenesis of the disease varies from population to population and is largely influenced by people’s perception of the disease.^6^ Hence it is important to identify the common factors within the population that present with RHD. There are no studies in more than 35 years in Uganda to document the characteristics of patients affected by RHD.

The symptoms of RHD depend on the valve lesion and its severity.^5^ Symptoms of RHD may not show for many years until valvular disease becomes severe. In general terms, initial symptoms of RHD are the symptoms of early heart failure: breathlessness on exertion, feeling tired and general weakness. As heart failure progresses, other symptoms may develop, including orthopnoea, paroxysmal nocturnal dsypnoea, and peripheral oedema. Palpitations may occur if atrial fibrillation is present (particularly in mitral stenosis). This arrhythmia is associated with increased thromboembolic risk, including stroke. People with aortic valve disease may experience angina and syncope in addition to heart failure symptoms. Clinical examination includes assessment of severity and complications, including signs of heart failure, the presence of atrial fibrillation and any new murmurs.

Early diagnosis of RHD is important as secondary prophylaxis can be started as soon as possible to help prevent the progression of valve disease.^5^ Echocardiography (echo) is essential to confirm the diagnosis and monitor the heart valves to detect any progression of disease.^7^,^8^ The management of RHD is complex and requires careful co-ordination. The main goal is to prevent disease progression and to avoid, or at least delay, valve surgery. Management of RHD depends on the severity of disease.^7^,^8^ The need for surgery is determined by the severity of symptoms, evidence that the heart valves are significantly damaged, the heart chamber size is distorted and cardiac function is significantly impaired.^9^

To our knowledge, there are no systematically collected data on newly diagnosed patients with RHD in the Ugandan setting over the past 30 years. Accordingly, the aim of this study was to describe the presenting features and complications of patients who were newly diagnosed with RHD in the Mulago Hospital.

## Methods

Institutional ethics approval was obtained from the School of Medicine Research and Ethics Committee of the College of Health Sciences, Makerere University. We obtained informed consent from all patients and informed assent for those unable to give consent. Patients’ initials and study numbers were put on the questionnaires instead of full names to ensure confidentiality.

This was a cross-sectional study describing the clinical and echo features of newly diagnosed RHD patients between June 2011 and January 2012. The study was carried out at Mulago Hospital, the national referral hospital and Makerere University’s teaching hospital, located in Kampala, Uganda. Mulago Hospital handles about 25 patients with newly diagnosed RHD a month at different clinics, as follows: (1) Uganda Heart Institute located on ward 1C, Mulago Hospital (in- and out-patient departments) registers on average 10 newly diagnosed RHD patients per month;^10^ (2) The adult cardiac clinic in the medical out-patient department (MOPD), Mulago Hospital registers six newly diagnosed RHD patients per month; (3) The paediatric cardiac clinic in the MOPD, Mulago Hospital registers about one newly diagnosed RHD patient per month; (4) The cardiac in-patient firm (ward 4C), Mulago Hospital admits about eight newly diagnosed RHD patients per month.

RHD cases were diagnosed using the WHO and United States National Institutes of Health-recommended echo diagnostic criteria.^11^ Complications of RHD were defined as one or more of the following: (1) advanced heart failure (NYHA class III/IV), (2) atrial fibrillation, (3) infective endocarditis (diagnosed using the modified Duke criteria), (4) pulmonary hypertension, (5) atrial thrombus, (6) thromboembolic stroke secondary to atrial fibrillation or infective endocarditis, (7) recurrent ARF (diagnosed using NIH/WHO criteria).^11^ The inclusion criteria were age five to 65 years of age in newly diagnosed RHD patients, confirmed by echocardiography (echo) using the above criteria.^11^ Patients with prior echo diagnosis of RHD were excluded.

**Fig. 1.**
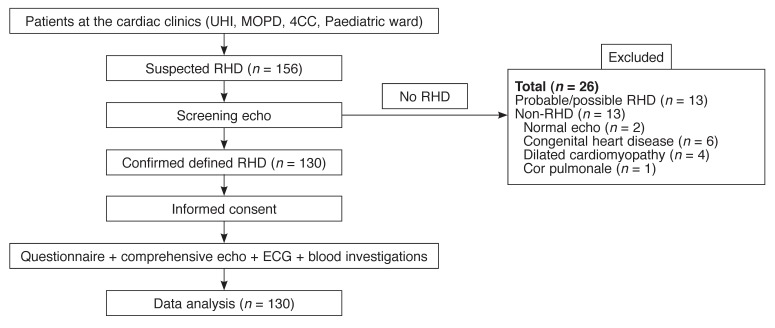
Patients’ flow chart. UHI = Uganda Heart Institute; MOPD = Medical out-patient department; 4C = Ward 4C cardiology

Patients who met the inclusion criteria were consecutively recruited (Fig. 1) over a period of eight months to reach the required sample size of 130 patients. Data on demographic variables (age, gender, tribe, residence, occupation, income level, education level) and clinical variables (history, physical examination, laboratory investigation variables, rest ECG, echo) were recorded on a standardised questionnaire.

## Transthoracic echocardiography (TTE)

A commercially available cardiac ultrasound machine, Vivid 7 Dimension, GE Medical Systems (Horten, Norway) with dedicated capabilities for cardiac evaluation, was used to acquire the images. Image acquisition was performed according to the ASE guidelines.^12^ Briefly, transthoracic echocardiograms were performed with the subjects at rest in the left lateral decubitus position by the principle investigator, under supervision of an experienced cardiologist. The recorded images were reviewed by two independent experienced cardiologists who did not know the patients. A 3.5-MHz transducer was used for adult (age > 12 years) two-dimensional, M-mode and Doppler examinations, and a 5.0–7.5-MHz transducer was used for children (age 5–12 years).

## M-mode and two-dimensional echocardiography

Simultaneous M-mode and two-dimensional echocardiography was performed. M-mode recordings were made in the parasternal long-axis (PLAX) position during apnea with the cursor at the level of the chordae tendineae and papillary muscles. PLAX, parasternal short-axis (PSAX), apical four-chamber (A4C) and apical two-chamber (A2C) views were taken in ciné-loop format and recorded on DVD discs in both DICOM and MPEGVUE for subsequent evaluation by the independent team. An echocardiography report was written according to the laboratory’s protocol and handed to the patient.

Severity of the valvular lesion was classified according to the ACC/AHA.^13^ Left ventricular and left atrial dilatation were defined as left ventricular diastolic diameter and left atrial diameter more than 57 and 40 mm, respectively.^13^ Left ventricular systolic dysfunction was defined as ejection fraction less than 55%.^13^ Pulmonary artery systolic pressure (PASP) was estimated from the peak velocity of the tricuspid regurgitation jet plus the estimated right atrial pressure. Patients with PASP ≥ 30 mmHg were classified into mild (< 50 mmHg), moderate (50–79 mmHg) and severe (≥ 80 mmHg) pulmonary hypertension.^13^

## Statistical analysis

Data were captured into EPI-DATA (version 3.1), cleaned and then exported to Stata version 10 for analysis. Continuous variables were summarised as mean (± standard deviation) and median (inter-quartile range), and presented in the tables. Categorical data were analysed using frequency and percentages, and results are presented in frequency tables and bar charts. Test of significance (*p*-value) was determined using the chi-square test. A *p*-value of less than 0.05 was considered significant.

## Results

We screened over a period of eight months, 156 patients who were suspected clinically of having RHD, using the echo machine. Twenty-six patients were excluded for the following reasons: probable/possible RHD (13 cases), normal echo findings (two cases), congenital heart disease (six cases), dilated cardiomyopathy (four cases) and cor pulmonale (one case); 130 patients who were confirmed to have definite RHD were recruited and entered in the data analysis (Fig. 1).

Table 1 shows the demographic characteristics of the 130 newly diagnosed cases of RHD. Overall, females (72.3%) predominated, with a younger median age of males than females (24 vs 33 years). The majority of the study population’s highest education level was primary school (total: 46.2%; male: 52.8%; female: 43.6%), while 10% (male: 8.3%; female: 10.6%) were illiterate. Unemployment rate was as high as 64.6% (male: 52.8%; female: 69.2%) and 32.3% (male: 44.4%; female: 27.7%) lived in temporary houses.

**Table 1:** Socio-Economic Data Of Newly Diagnosed Rheumatic Heart Disease Patients (*n* = 130)

	*All (n = 130)*	*Females (n = 94)*	*Males (n = 36)*
Gender distribution (%)	100	72.3	27.69
Median age (years)	29.5	33	24
Educational level			
none, *n* (%)	13 (10)	10(10.64)	3(8.33)
primary, *n* (%)	60 (46.15)	41 (43.62)	19 (52.78)
secondary, *n* (%)	42 (32.31)	31 (32.98)	11 (30.56)
college/university, *n* (%)	16 (12.31)	8 (8.51)	8 (22.22)
No formal employment	84 (64.62)	65 (69.15)	19 (52.78)
Temporary housing	42 (32.31)	26 (27.66)	16 (44.44)

**Fig. 2.**
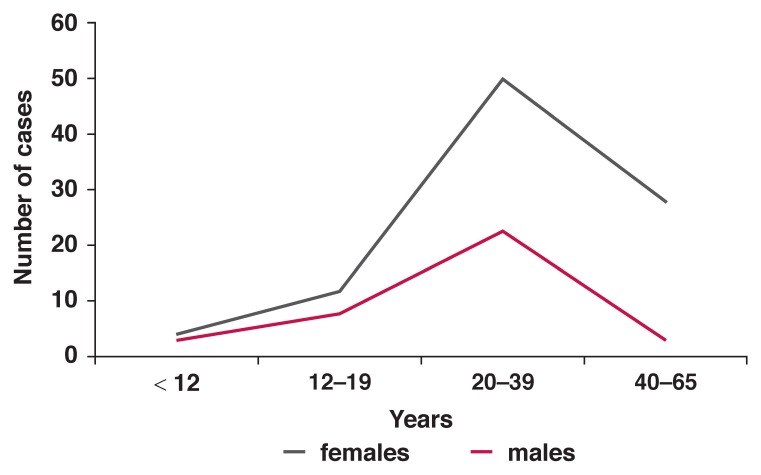
Age distribution of newly diagnosed RHD.

The age distribution of newly diagnosed RHD patients showed a peak in the young adult age group (20–39 years). The disease was lowest in the age group < 12 years (5.4% of RHD cases), increased in the 12–19-year group (15.4%), peaked at 20–39 years (55.4%), followed by a declining number of case presentations in the age group 40–65 years (23.8%). The pattern of case presentation according to age was similar for males and females (Fig. 2).

**Fig. 3.**
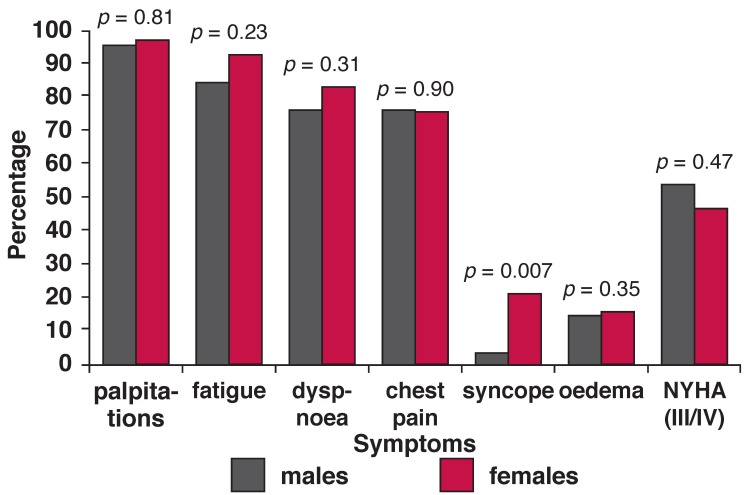
Frequncy of symptoms in newly dignosed patients.

Fig. 3 shows the frequencies of symptoms with which the study participants presented. Palpitations were the commonest symptom (95.4%), followed by fatigue (89.2%) and dyspnoea (75%). Other symptoms included chest pain (74.6%), syncope (15.4%) and oedema (14.6%). There were no gender-specific statistical differences in most of the symptoms, except females reported more syncope than males (20.2 vs 2.8%) and more males presented with severe heart failure than females.

Table 2 shows the frequency distribution of rheumatic valve lesions by age group. Isolated or multiple valve lesions were observed in the spectrum of RHD. There were eight types of valvular lesions detected according to the valve affected. Pure mitral regurgitation (MR) was the most prevalent lesion (55 cases, 42.3%), followed by MR + aortic regurgitation (AR) (36 cases, 27.7%). Other lesions included MR + plus mitral stenosis (MS) + AR (11 cases, 8.5%), MR + MS (nine cases, 6.9%), pure MS (nine cases, 6.9%), MS + AR (seven cases, 5.4%), MR + MS + AR + aortic senosis (AS) (two cases, 1.5%) and MS + AS + AR (one case, 0.8%).

**Table 2:** Distribution Of Valve Lesion By Age Group

	*All*	*<12 years*	*12–19 years*	*20–39 years*	*40–65 years*
*Valve lesion(s)*	130 (100%)	7	20	72	31
MR	55 (42.31)	4 (57.14)	12 (60)	26 (36.11)	12 (38.71)
MS	9 (6.92)	0	0	7 (9.72)	2 (6.45)
MR + MS	9 (6.92)	0	1 (5)	6 (8.33)	2 (6.45)
MR + AR	36 (27.69)	3 (42.86)	5 (25)	20 (27.78)	8 (25.81)
MS + AR	7 (5.38)	0	0	4 (5.56)	3(9.68)
MR + MS + AR	11 (8.46)	0	1 (5)	6 (8.33)	4 (12.90)
MS + AS + AR	1 (0.78)	0	0	1 (1.39)	0
MR + MS + AR + AS	2 (1.54)	0	0	2 (2.78)	0

MR = mitral regurgitation; MS = mitral stenosis; AR = aortic regurgitation; AS = aortic stenosis.

The mitral valve was involved in all cases, while 73 cases (56.2%) had isolated mitral valve lesions. All patients who had aortic valve disease had associated mitral valve disease (57 cases, 43.8%). Only one case was found to have abnormal morphology (thickening) of the tricuspid valve. Isolated MR or in association with AR was the most common finding detected among children and adolescents (100% of age group < 12 years and 85% of 12–19 years). MS was less frequent in these age groups. Although MS, AS and multiple valve lesions appeared in adolescents, their frequency increased in young adult patients.

**Fig. 4.**
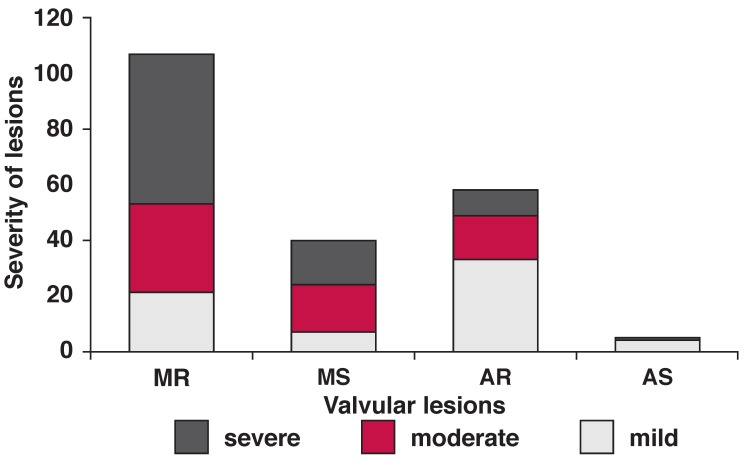
Severity of valve lesions.

Fig. 4 shows the degree of severity of RHD according to valvular lesion. The four types of valvular lesions were found in mild, moderate and severe forms; 72.9% of the lesions fell into the moderate and severe degree. Moreover, in mild-degree lesions, AR prevailed, while in the severe form, MR was predominant.

Table 3 shows the echo features and complications of the study population according to the predominant rheumatic valvular lesion. Patients having haemodynamically significant valvular disease affecting two valves were counted twice. Presentation with an impaired systolic function of a left ventricular ejection fraction < 55% was not uncommon; 20 of 112 cases (17.9%) presenting with mitral regurgitation and five of 28 cases (17.9%) presenting with aortic regurgitation.

**Table 3:** Echocardiographic Features And Complications According To Predominant Valvular Lesion

*Total cases*	*Total 130 (100%)*	*MS 30 (23.08%)*	*MR 112 (86.15%)*	*AS 1 (0.77%)*	*AR 28 (21.15%)*
Echo features					
Mean LVEF	61.15	63.96	59.85	67	48.76
Systolic dysfunction	27 (20.77)	2 (6.67)	20 (17.86)	0	5 (17.86)
Mean LVIDD	55.89	43.9	57.57	55	62.69
LV dilatation	60 (46.15)	3 (10)	60 (53.57)	0	15 (53.57)
Mean LA	50.48	52.02	49.35	50	50.46
LA dilatation	98 (75.38)	25 (83.33)	78 (69.64)	1 (100)	24 (85.71)
Spontaneous echo contrast	3 (2.31)	1 (3.33)	2 (1.79)	0	2 (7.14)
Complications					
PHT	73 (56.15)	16 (53.33)	56 (50)	0	13 (46.43)
IE	10 (7.69)	1 (3.33)	8 (7.14)	0	3 (10.71)
AF	18 (13.85)	7 (23.33)	11 (9.82)	0	2 (7.14)
NYHA class III/IV	56 (43.08)	13 (43.33)	42 (37.5)	0	9 (32.14)
Definite recurrent ARF	4 (3.08)	1 (3.33)	3 (2.68)	0	0
Probable recurrent ARF	4 (3.08)	0	4 (3.57)	0	0

LV = left ventricle; LVEF = left ventricular ejection fraction; LVIDD = left ventricular internal diameter in diastole; LA = left atrium; PHT = pulmonary hypertension; IE = infective endocarditis; AF = atrial fibrillation; NYHA = New York Heart Association; ARF = acute rheumatic fever.

Chamber dilatations were much more frequent findings than systolic dysfunction; left ventricular dilatation was seen in 60 (46.2%) cases, with a predominant proportion with MR (53.6%) and AR (56.2%). Prevalence of left atrial dilatation in the study group was as high as 75.4%. Pulmonary hypertension (53.3%) was the most detected complication by echo and more related to mitral lesions (both MR and MS). Other complications were less frequent. Atrial fibrillation (13.9%) was the characteristic complication of MS. Infective endocarditis was found in 10 (7.7%) cases, and was mainly associated with MR and AR.

Although no patient presented with a history of stroke or left atrial thrombus in our study group, three of the patients had echo findings of left atrial spontaneous echo contrast, which carries a very high risk of left atrial thrombus formation and cardiovascular accident (stroke). Almost half of the patients presented with clinical heart failure in NYHA class III and IV. This was more prevalent in patients with mitral valve lesions. Eight (6.2%) patients had evidence of recurrent ARF and 93 cases (71.5%) required valvular surgery, according to the NHFA/CSANZ 2006 guidelines of management of RHD.^9^

## Discussion

In this small, tertiary hospital-based study, we described the presenting features and complications of newly diagnosed RHD patients in a Ugandan population. All participants were indigenous blacks and 72.3% of the study participants were female, which concurs with the Soweto study where 68% were female.^14^ It contrasts with the Pakistan study were only 46% were female.^15^ More males had formal education than females. Lack of formal employment was more prevalent in females than males. The rates of living in temporary housing were similar in both genders.

Although this study did not evaluate the association between socio-economic status and RHD presentation, the finding that low levels of formal education, high levels of unemployment and poor housing conditions underscored their role in determining disease incidence.^16^ On the other hand, the nature of the heart disease will have an impact on an individual’s education and employment opportunities. Hence, there might be a vicious circle between socio-economic status and RHD in the population. This reminds us that control of the disease needs a dual effort from both the economic sector and health service systems.

The higher prevalence of disease in females than males correlated with their illiteracy and unemployment status. However, this could have been attributed to factors such as genetic predisposition, hormonal factors and poor health-seeking behaviours among males. This needs to be studied further.

Fatigue and palpitations were the most common presenting symptoms, followed by difficulty in breathing and chest pain. Given that fatigue and palpitations are non-specific symptoms of many physiological and pathological conditions, including early heart failure,^5^ it is proposed that health workers do not overlook these symptoms. Improvement in disease awareness at the community level is needed in order to diagnose the disease as early as possible. The finding that over 40% of patients presented in NYHA class III/IV indicates the poor quality of life, delayed diagnosis and low level of knowledge of the disease in the population, among both patients and health workers.^17^,^18^

We found that the most common lesions seen in patients with newly diagnosed RHD were pure MR, followed by MR + AR. Tricuspid valve involvement was extremely rare. Regurgitation was more common than stenotic lesions. Stenotic lesions were understandably rare in children and adolescents, as time is required for fibrosis and re-organisation to develop. Multiple valvular lesions were mainly seen in young adults. This finding is very important. For example, the finding that the most common multi-valve lesion was MR + AR, and it was most prevalent in the age group 20–39 years supports available evidence that repeated attacks of ARF in RHD patients are responsible for disease progression, thus underscoring the importance of prophylaxis against repeated ARF.

Most valvular lesions in the patients were in the moderate-to-severe form, which is consistent with previously reported data from different countries.^17^-^20^ Beaton and colleagues have previously reported 4.9 cases of mild RHD per 1 000 asymptomatic school children in Uganda.^21^ This finding, combined with the finding that predominant disease in the hospital was moderate to severe, again reinforces the importance of screening and regular echocardiographic checks for high-risk populations. Early intervention with prophylaxis would protect other valves from infection and also control the progression of the affected valve(s).

There were 6.9% of patients who had pure MS, and 6.9% had MS + AR. The majority of these patients were in the age group 20–39 years. These patients could benefit from percutanous mitral valvoplasty, which has been available at the Uganda Heart Institute since December 2012. Optimal benefit depends on early presentation before calcification and development of other complications,^22^ such as gross atrial dilatation, atrial fibrillation and severe CCF, further emphasising the need for early disease detection.

Almost half (43.1%) of the patients presented in NYHA class III/IV heart failure, but 20.8% of patients had a calculated ejection fraction (EF) of less than 55%. The lowest mean EF in AR cases was related to the finding that AR was associated with the most dilated left ventricles, understandably due to volume overload and compensatory left ventricular (LV) wall stretch (Table 3). All disease categories presented with significant dilatation of the left atrium (LA). This frequency of LA dilatation could partly explain the high prevalence of pulmonary hypertension (PHT). Atrial fibrillation was more frequent in MS and MR.

The presence of these complications heavily influences the method and outcome of treatment, including surgery where possible.^23^ Patients with gross distortions of the heart, notably grossly dilated atria will require chamber resection during valve replacement.^23^ This makes the operation more expensive but also increases the risk of postoperative complications. Patients with atrial fibrillation will need warfarin for prophylaxis against thromboembolism. This however is associated with a high risk of bleeding due to difficulty in INR monitoring and control, as most patients are too poor to afford the cost of the test.

The data from this study showed a predominant proportion of young adults with advanced forms of RHD, which is in agreement with findings from other studies in Africa.^14^,^18^ We also noticed a 6.2% of recurrent ARF among our study group, which was similar to that described in the Fiji study,^22^ however none of the study population could recall a clear history of past ARF. This reaffirms the importance of meticulous secondary prophylaxis. That the majority of patients were in the age group 20–39 years reflects the adolescent nature of ARF/RHD. The presentation by the majority with moderate-to-severe disease confirms the poor/low diagnostic rate of ARF in this population.

The findings of this study indicate that the majority of patients with RHD present with palpitations, fatigue, dyspnoea and chest pain. Given that these are not specific symptoms for RHD, it is important for general practitioners and other lowercardre health professionals who see the majority of these patients to suspect RHD and refer these patients for specialist evaluation using echocardiography. This would facilitate early confirmatory diagnosis of cases and hence aid in early intervention, so preventing complications.

Our study had a number of limitations. First, this study population reflects those who were fortunate enough (or sick enough) to seek specialist care at the hospital and was always likely to describe those with more advanced forms of RHD. Second, unfortunately, there are no gold-standard diagnostic criteria for RHD. We applied a clinically orientated approach based on published criteria and acknowledge that there may be inherent biases in our classification of cases. For example, according to the NIH/WHO RHD echo-diagnosing criteria,^11^ an isolated aortic valvular lesion is not considered a definite RHD case. That is why there was no patient with isolated aortic vulvlar lesion seen in our study.

## Conclusion

Rheumatic heart disease continues to be a major health problem in cardiac patients presenting to Mulago Hospital. It accounts for a large percentage of cardiovascular disease-related admissions and is an important indication for cardiac surgery in Uganda. Patients with newly diagnosed RHD in Mulago present with an advanced disease pattern of clinically severe symptoms associated with poor quality of life, moderate-to-severe form of valvular lesions and high frequency of complications. All these reflect a high burden of RHD in this country, a delayed diagnosis and delayed seeking of medical services. Young females accounted for the majority of the study population. The majority of the newly diagnosed RHD patients required valvular heart surgery, which is not yet available locally.
